# Deletion of 1.8-kb mRNA of Marek's disease virus decreases its replication ability but not oncogenicity

**DOI:** 10.1186/1743-422X-7-294

**Published:** 2010-10-29

**Authors:** Aijun Sun, Yanpeng Li, Jingyan Wang, Shuai Su, Hongjun Chen, Hongfei Zhu, Jiabo Ding, Zhizhong Cui

**Affiliations:** 1 Animal Science and Technology College, Shandong Agricultural University, Tai'an, Shandong 271018, China; 2 The Institute of Animal Science, Chinese Academy of Agricultural Sciences, Beijing 100193, China; 3 Shanghai Veterinary Research Institute, Chinese Academy of Agricultural Sciences, Shanghai, 200241, China; 4 China Institute of Veterinary Drug Control, Beijing 100081, China

## Abstract

**Background:**

The 1.8-kb mRNA was reported as one of the oncogenesis-related genes of Marek's disease virus (MDV). In this study, the bacterial artificial chromosome (BAC) clone of a MDV field strain GX0101 was used as the platform to generate mutant MDV to examine the functional roles of 1.8-kb mRNA.

**Results:**

Based on the BAC clone of GX0101, the 1.8-kb mRNA deletion mutant GX0101Δ(A+C) was constructed. The present experiments indicated that GX0101Δ(A+C) retained a low level of oncogenicity, and it showed a decreased replication capacity in vitro and in vivo when compared with its parent virus, GX0101. Further studies in vitro demonstrated that deletion of 1.8-kb mRNA significantly decreased the transcriptional activity of the bi-directional promoter between 1.8-kb mRNA and pp38 genes of MDV.

**Conclusion:**

These results suggested that the 1.8-kb mRNA did not directly influence the oncogenesis but related to the replication ability of MDV.

## Background

Marek's disease (MD) is a contagious lymphoproliferative disease of poultry caused by the highly oncogenic alphaherpesvirus, MDV, which is characteristic by mononuclear infiltration of peripheral nerves, irises, skin and other visceral tissues [1,2]. Among the 100 genes encoded by MDV, three genes including 1.8-kb mRNA, pp38 and meq were considered to be associated with oncogenicity of MDV serotype 1, and they are also unique to MDV [3,4]. Previous studies suggested that meq is involved in lymphocyte transformation [5,6], and pp38 is involved in early cytolytic infection in lymphocytes but not in the induction of tumors [7]. In addition, recent studies indicated that pp38 could also enhance the activity of the bi-directional promoter, which locates between pp38 and 1.8-kb mRNA in the long inverted repeat region of the viral genome, thus influence the replication capacity of the virus [8-10].

The 1.8-kb mRNA is unique to MDV and it has no homology with other groups of herpesviruses, and it received attention as a pathogenic determinant following demonstration of the expansion of the 132-bp tandem repeats in the 1.8-kb mRNA region during attenuation of MDV. However, deletion of the two copies of the 132-bp repeat region in a pathogenic MDV demonstrated that the virus was still pathogenic [11]. The transcription map of 1.8-kb mRNA was published in 1989 [12], analysis of cDNA in the 1.8-kb mRNA region identified two main open reading frames (ORFs) (ORF A and ORF C), and the proteins encoded by ORF A and C could be detected in chicken embryo fibroblasts (CEF) infected with very virulent MDV as well as MDV-induced lymphoid cell lines [13]. Therefore, in the present study, ORF A and C were selected as the targets to study.

Recent progresses in BAC cloning and mutagenesis technology make it possible to identify specific genes important for MDV replication and oncogenesis. In earlier studies we cloned the full length genome of a virulent MDV strain, GX0101, into a bacterial artificial chromosome (BAC) and reconstituted the infectious virus, bac-GX0101. Studies in specific-pathogen-free (SPF) chickens showed that the virulence of bac-GX0101 was higher than virulent MDV (vMDV) GA strain but lower than very virulent MDV (vvMDV) strain Md5, and there was no difference in growth ability and pathogenicity to birds when compared with its parental virus, GX0101 [14-16]. In this study, the BAC clone of GX0101 was used as the platform to generate mutant MDV to examine the functional roles of 1.8-kb mRNA.

## Results

### Verification of GX0101Δ(A+C)

**Figure 1:**
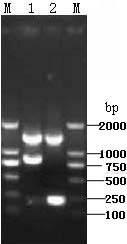
**Analysis of PCR products of GX0101Δ(A+C)-BAC DNA**. Lane M: DL2000 marker (TaKaRa Bio-Company, China); 1: the PCR product of GX0101Δ1 (A+C); 2: the PCR product of GX0101Δ (A+C). The bigger band demonstrated one of ORF (A+C) was replaced by kana gene (in lanes 1 and 2). The smaller band demonstrated the deletion of the second ORF(A+C) in GX0101Δ(A+C)-BAC DNA (in lane 2) compared to the smaller band that not deleted the second ORF(A+C) in GX0101Δ1(A+C)-BAC DNA (in lane 1).

**Figure 2:**
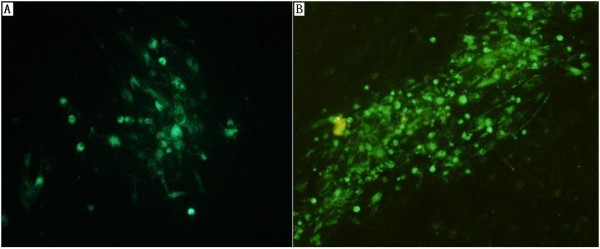
**Comparison of plaque characteristic of bac-GX0101Δ(A+C) and parental virus GX0101 in CEF**. A: plaque of GX0101Δ(A+C); B: plaque of GX0101. GX0101 and GX0101Δ(A+C) were inoculated onto six-well plates seeded with CEFs and incubated at 37°C, 5% CO_2_, respectively. Visible viral plaques were confirmed by IFA with monoclonal antibody H19. The plaque size of GX0101Δ(A+C) was smaller than that of GX0101 at 96 h after infected in fresh CEF cells.

The deletion of the ORF(A+C) was confirmed by PCR with purified GX0101Δ1(A+C)-BAC and GX0101Δ(A+C)-BAC as templates [16]. As shown in Figure 1, the deletion of both copies of ORF(A+C) was confirmed by agarose gel electrophoresis of PCR. Then the GX0101Δ(A+C)-BAC DNA was transfected into CEF for the rescue of GX0101Δ(A+C) virus. As shown in Figure 2, the plaque size of GX0101Δ(A+C) was smaller than that of GX0101 at 96 h after infected in fresh CEF cells.

### In vitro replication of GX0101Δ(A+C)

**Figure 3:**
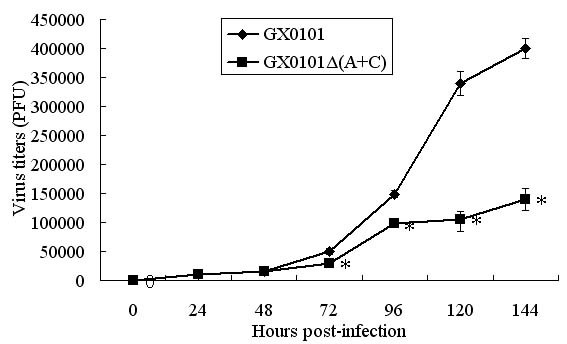
**Growth curves of GX0101Δ(A+C) and GX0101 in vitro**. 100 PFU GX0101 and GX0101Δ(A+C) were inoculated onto six-well plates seeded with 2×10^6 ^CEFs and incubated at 37°C, 5% CO_2_, respectively. At hours 0, 24, 48, 72, 96, 120 and 144 p.i., the infected cells were trypsinized and serial 10-fold dilutions were added onto six-well plates of CEFs, visible viral plaques were counted on days 5 p.i. by IFA. The means ± SD at each time point were shown, *P < 0.05 compared with those in GX0101 group. It was demonstrated that the mutant virus GX0101Δ(A+C) exhibited a decreased replication ability in CEF compared with GX0101 at hours 72, 96, 120 and 144 p.i. (P < 0.05).

To determine whether the deletion of the 1.8-kb mRNA had any effect on GX0101Δ(A+C) growth replication in vitro, the growth rate of GX0101Δ(A+C) virus was compared with that of GX0101. As shown in Figure 3, it was demonstrated that the recombinant virus GX0101Δ(A+C) exhibited a decreased replication ability in CEF compared with GX0101 at hours 72, 96, 120 and 144 post inoculation (p.i.) (P < 0.05).

### Viremia levels of birds infected with GX0101Δ(A+C) or GX0101 viruses

The viremia levels in 10 birds from each group were determined on days 7, 14, 21 and 28 p.i. As indicated in Table 1, the viremia levels of GX0101Δ(A+C) -infected group were lower than that of GX0101 group during the whole experimental period, and significant differences were observed between the two groups on days 14, 21 and 28 p.i. (P < 0.05).

**Table 1:** Comparision of viremia levels between GX0101 and GX0101Δ(A+C) infected SPF chickens (n = 10)

Days post inoculation	Viremia (PFU/ml)	
	
	GX0101	GX0101Δ(A+C)
7	156.5 ± 40.5 a	123.8 ± 27.3 a
14	475.8 ± 55a	255.0 ± 39.5 b
21	1567.4 ± 253.6 a	455.4 ± 98.7 b
28	1244.3 ± 242.6 a	513.4 ± 188.9 b

The numbers in the table indicate: mean ± standard deviation. The same letters following values indicate that the differences were not significant (P > 0.05) between treatments at each time. The different letters following values indicate that the differences were significant (P < 0.05) between treatments at each time.

### The effect of ORF (A+C) on the activity of bi-directional promoter

To determinate the activity of the bi-directional promoter, plasmids pP(pp38)-CAT and pP(1.8-kb)-CAT were used to transfect CEF monolayers. The results showed that the CAT expression level in uninfected CEF was very low, nearly 0. The CAT activity in GX0101-CEF was higher than that in GX0101Δ(A+C)-CEF (P < 0.05) (Table 2). The results indicated that 1.8-kb mRNA significantly affected the activity of the bi-directional promoter.

**Table 2:** Comparision of CAT expression levels under the promoter in opposite directions in GX0101Δ(A+C)-CEF or GX0101-CEF (n = 5)

Transfected plasmids	Transfected CEF		
	
	GX0101Δ(A+C)-CEF	GX0101-CEF	CEF
pP(pp38)-CAT	0.069 ± 0.013b	0.413 ± 0.045a	0.002 ± 0.0002c
pP(1.8-kb)-CAT	0.073 ± 0.024b	0.505 ± 0.068a	0.001 ± 0.0004c
Control	0.000 ± 0.000a	0.000 ± 0.000a	0.000 ± 0.000a

The numbers in the table indicate: mean ± standard deviation of five replicate assays with a given reporter plasmid. The same letters following values indicate that the differences were not significant (P > 0.05) between treatments. The different letters following values indicate that the differences were significant (P < 0.05) between treatments.

### Pathogenicity of GX0101Δ(A+C) and GX0101

To compare the pathogenicity of mutant virus with its parental virus, we examined the growth rates of infected birds. Both viruses strongly inhibited the growth rates of infected birds. As shown in Table 3, body weights of the birds inoculated with GX0101Δ(A+C) and GX0101 were significantly lower (P < 0.05) than that of control birds from 5 weeks p.i. Between the two viruses, GX0101 showed stronger effects on growth rates of birds than GX0101Δ(A+C) although the difference was not statistically significant (P > 0.05).

**Table 3:** Comparisons of growth rates of birds challenged with GX0101Δ(A+C) or GX0101

Weeks post inoculation	Groups		
	
	Control	GX0101Δ(A+C)	GX0101
3	117.91 ± 9.40 (13) a	111.62 ± 18.62 (34) a	117.88 ± 17.63 (33) a
4	189.17 ± 18.44 (13) a	175.74 ± 33.62 (34) a	174.84 ± 32.44 (31) a
5	275.42 ± 30.34 (13) a	243.24 ± 46.9 (34) b	233.83 ± 51.82 (30) b
6	401.25 ± 41.24 (13) a	335.61 ± 62.71 (33) b	309.66 ± 91.74 (29) b
8	672.92 ± 72.53 (13) a	571.72 ± 102.58 (32) b	510.19 ± 155.27 (26) b

The numbers in the table indicate: mean ± standard deviation. Different letters indicate that the differences were significant (P < 0.05) between treatments at each time.

During 120 days after challenged with the two viruses, 50% and 40% mortality were observed in groups inoculated with GX0101 or GX0101Δ(A+C), respectively. Furthermore, 22.5% and 12.5% of birds exhibited visceral tumors conformed by histopathologic changes in different tissues (spleen, liver, heart, and kidney) in groups infected with GX0101 or GX0101Δ(A+C), respectively (Table 4). And no death was observed in the control group. These results showed that the mortality and oncogenicity of GX0101Δ(A+C) were lower than that of GX0101, although the difference was not significant (P > 0.05).

**Table 4:** Comparison of the mortality and oncogenicity in SPF chickens

Strain	Mortality (%)	Oncogenicity (%)
GX0101	20/40 (50.0)	9/40 (22.5)
GX0101Δ(A+C)	16/40 (40.0)	5/40 (12.5)
Control	0/13 (0)	0/13 (0)

### Immunosuppressive effects of the two viruses

As demonstrated in Table 5, hemagglutination inhibition (HI) antibody titers to AIV-H9 in birds infected with GX0101Δ(A+C) or GX0101 were significantly lower than that of the control birds (P < 0.05). Between the two viruses, HI antibody titers to AIV-H9 in birds infected with GX0101 were significantly lower than that of birds infected with GX0101Δ(A+C) (P < 0.05). However, HI antibody titers to AIV-H5 and NDV, GX0101 showed stronger immunosuppressive effects than GX0101Δ(A+C), although the difference was not statistically significant (P > 0.05).

**Table 5:** Influence of GX0101Δ(A+C) and GX0101 virus infections on HI antibody titers to NDV, AIV-H5 and AIV-H9 after vaccination

Strain	HI titers (log2)		
	
	NDV	AIV-H5	AIV-H9
GX0101	9.71 ± 1.46(28)b	4.45 ± 2.92(28)b	3.48 ± 2.06(28)c
GX0101Δ(A+C)	10.07 ± 1.23(33)a	5.63 ± 2.77(33)b	5.50 ± 2.39(33)b
Control	10.57 ± 0.76(13)a	7.15 ± 1.41(13)a	7.00 ± 1.41(13)a

The numbers in the table indicate: mean ± standard deviation. Different letters indicate that the differences were significant (P < 0.05) between treatments.

## Discussion

It was reported that CAT activity under the control of the bi-directional promoter was only detected in MDV-infected CEF but not in uninfected CEF when transfected with CAT reporter plasmids, indicating that the bi-directional promoter requires either viral or MDV-infection related cellular factors for regulation [17]. In the previous reports, we found that the activity of bi-directional promoter in the direction of the 1.8-kb mRNA was higher than that in the direction of the pp38, and the CAT activity was significantly lower but not disappeared in a pp38 deletion virus than in the parental virus [8-10]. This suggested that pp38 plays an important role in regulating the transcriptional activity of the bi-directional promoter, but that an additional factor may also be necessary. In this study, CAT gene was used as a reporter to investigate the influence of 1.8-kb mRNA on its upstream bi-directional promoter. The results showed that the CAT expression level of GX0101Δ(A+C)-CEF was significantly lower than that in GX0101-CEF transfected with two CAT reporter plasmids under the control of the bi-directional promoter in two opposite oppositions. These results suggested that 1.8-kb mRNA was necessary in addition to pp38 for transcriptional activity of the bi-directional promoter. However, either pp38 or 1.8-kb mRNA did not fully affect the promoter activity, respectively. Our future studies will focus on the construction of pp38- and 1.8-kb mRNA-deleted virus, it may help to examine whether the activity of the promoter will be fully removed after both pp38 and 1.8-kb mRNA were deleted.

Following deletion of ORF(A+C), we found that the replication ability of GX0101Δ(A+C) was decreased compared to that of GX0101 in vitro and in vivo. MDV replication origin locates between pp38 and 1.8-kb mRNA in the long inverted repeat region of the viral genome, therefore, we speculate that 1.8-kb mRNA might affect the MDV replication origin as its effect to the bi-directional promoter. However, further studies are required to confirm this hypothesis and to understand the mechanism of 1.8-kb mRNA.

Although GX0101Δ(A+C) was severely impaired for in vivo replication, the virus retained a low level of oncogenicity and thus demonstrated that 1.8-kb mRNA was dispensable for tumor induction. Meanwhile, HI antibody titers to AIV-H9, AIV-H5 and NDV in birds infected with GX0101 were lower than those infected with GX0101Δ(A+C) (P < 0.05). The differences in tumor induction or immunosuppression effects between GX0101Δ(A+C) and GX0101 may be due to the poor replication ability in vivo of GX0101Δ(A+C).

## Conclusion

These results suggested that the 1.8-kb mRNA gene family did not directly influence the oncogenesis of MDV but related to its replication ability. And our future studies will concentrate on the identification of the 1.8-kb mRNA product and its mechanism.

## Methods

### Virus and Plasmid

A field virulent MDV strain, named GX0101, was isolated from a layer farm in Guangxi province in China [14]. In our previous study, a full-length infectious BAC clone of GX0101 strain and Escherichia coli EL250 (harboring the GX0101-BAC containing the whole genome of GX0101) were constructed [15,16].

The recombinant plasmids expressing chloramphenicol acetyltransferase (CAT) gene under the control of the bi-directional promoter were constructed in our laboratory [9]. In the recombinant plasmid pP(pp38)-CAT, CAT was expressed under the promoter in pp38 direction, and in the recombinant plasmid pP(1.8-kb)-CAT, CAT was expressed under the promoter in 1.8-kb direction.

### Construction of GX0101Δ(A+C)-BAC

Replacement of the ORF (A+C) with the Kan^R ^gene was carried out by a procedure of homologous recombination [16]. Electrocompetent cells were prepared from *Escherichia coli *EL250 (harboring the GX0101-BAC) grown at 30°C in Luria-Bertani (LB) medium containing chloramphenicol (25 mg/ml) to an optical density (OD600) of 0.6. The expression of recE, recT, and λ gam was induced by 42°C for 15 min. Kan^R ^cassette flanked by FRT sites was amplified using primers 1.8-kb mRNA (A+C)-kan^R^-F and 1.8-kb mRNA(A+C)-kan^R^-R (Table 6) from pKD13 [18]. About 300 ng of the PCR products were electroporated into 50 μl of electrocompetent EL250 cells harboring the GX0101-BAC using standard electroporation parameters (2.0 KV, 200 Ω and 25 μF), and recombinant clones were isolated and examined for insertion of kan^R ^into the right locus using PCR. Once individual clones were examined and confirmed to lack spurious changes, kan^R ^was excised by induction of FLP recombination by incubation in LB medium containing 0.02% arabinose for 12 h. In order to delete the second ORF(A+C) copy, the entire procedure was repeated. Once both copies were deleted, an recombinant virus, named GX0101Δ(A+C), was reconstituted by transfecting CEF cultures with purified BAC DNA. And the recombinant virus, in which one copy ORF(A+C) was deleted, named as GX0101Δ1(A+C).

**Table 6:** List of primers used for the deletion of ORF A and C

Primers	Sequence 5'-3'
1.8-kb mRNA(A+C)-kan^R^-F	5'-aaaggatctcaattaatagaacggcgattttttatttacggcgatatttg CGTGTAGGCTGGAGCTGCTTC^*a*^-3'
1.8-kb mRNA(A+C)-kan^R^-R	5'-aaacagtttctaatcgaaagcgttaccgaacttgtctttaatgagaatcc CATTCCGGGGATCCGTCGAC^*a*^-3'

^*a *^For primers 1.8-kb(A+C)-kan^R^-F, 1.8-kb(A+C)-kan^R^-R, underlined sequences indicate the sequences from pKD13 used to amplify the Kan^R ^gene cassete with FRT, and sequences in bold indicate MDV sequence flanking the ORF (A+C).

### Confirmation of the deletion of ORF(A+C)

The deletion of the ORF(A+C) from the GX0101 BAC DNA were analyzed by PCR using primers, which cross the 1.8-kb mRNA ORF(A+C), as follows: forward primer, 5'-GGCTAGCATTCGATAAGC-3'; reverse primer, 5'-GGAGGTGTAATATAAGG G-3'. GX0101Δ(A+C) was reconstituted by transfecting CEF cultures with purified BAC DNA as previously described [15]. When CEF started to show plaques, the cells were passed by trypsinization. The virus-containing cells were passed 3 to 4 rounds for enrichment of infectious clone virus GX0101Δ(A+C). Finally, the cells infected with GX0101Δ(A+C) were harvested and stored in liquid nitrogen.

After transfection of the mutant viruses into CEF cultures, we compared the plaques of GX0101Δ(A+C) with those of GX0101 by immunofluorescence analysis (IFA) with monoclonal antibody H19 at 96 h after infected in fresh CEF cells as previously described [19] with modifications. Briefly, infected cells were washed with PBS and fixed with ethanol:acetone solution (4:6) at room temperature for 10 min. After removing fixing solution, the cells were air dried, and incubated with H19 (1:1000) for 1 h at 37°C. Following three washes with PBS, the cells were incubated with goat anti mouse FITC labeled secondary antibodies (Sigma) for 1 h. Cells were further washed three times with PBS and examined under a fluorescence microscope.

### In vitro and in vivo replication of GX0101Δ(A+C)

The rates of growth in vitro of GX0101Δ(A+C) were measured as follows, briefly, 100 PFU GX0101 and GX0101Δ(A+C) (from stock virus in liquid nitrogen) were inoculated onto six-well plates seeded with 2×10^6 ^CEFs and incubated at 37°C, 5% CO_2_, respectively. At hours 0, 24, 48, 72, 96, 120 and 144 p.i., the infected cells were trypsinized and serial 10-fold dilutions were added onto six-well plates of CEFs, visible viral plaques were counted on days 5 p.i. by IFA.

In vivo replication of GX0101Δ(A+C) were measured as follows. In brief, blood samples in anticoagulants were collected from 10 birds of each group on days 7, 14, 21 and 28 p.i., and 1 ml blood from each bird was mixed with 9 ml DMEM. Blood suspensions were centrifuged for 5 min at 500 g to separate white blood cells. And 500 μl white blood cells (10^6^cells/ml) collected after centrifugation were used to inoculate two duplicate 35-mm plates with CEF monolayer. To determine viremia levels, visible viral plaques were counted on days 5 p.i. by IFA.

### Determination of CAT activity in GX0101-CEF and GX0101Δ(A+C)-CEF

To analyze the transcriptional activity of the bi-directional promoter in GX0101Δ(A+C) infected CEF, plasmids pP(pp38)-CAT and pP(1.8-kb)-CAT were used to transfect GX0101-CEF or GX0101Δ(A+C)-CEF, respectively. Construction of recombinant plasmids expressing CAT gene under the control of the bi-directional promoter was performed as previously described [9]. Briefly, the bi-directional promoter sequences were amplified by PCR, and the PCR products were inserted into pCAT-Basic vector (Promega) at the KpnI and SacI sites. In the recombinant plasmids, pP(pp38)-CAT and pP(1.8-kb)-CAT, CAT was expressed under the regulation of the promoter in opposite directions. Transfection of recombinant plasmid DNA was performed as previously described, and all dishes were incubated at 37°C in a CO_2 _incubator [9]. The expression of CAT was determined 48 h after transfection. The transfected CEF were harvested and resuspended in 500 μl lysis buffer per 35 mm dish, and samples were centrifuged for 5 min at 10,000 rpm. Aliquots of the supernantants were detected with CAT ELISA Kit (Roche).

### Pathogenicity of GX0101 and GX0101Δ(A+C)

One-day-old male SPF chickens were randomly divided into three groups and kept in three isolators under positive filtered air. In the experiment, 40 birds were inoculated intra-abdominally with 1000 PFU of GX0101 or GX0101Δ(A+C). A control group of 13 birds was inoculated with uninfected CEF. During 120 days after challenge with the two viruses, all chickens were examined for gross MD lesions. Body weight measurements of the birds in different groups were made on weeks 3, 4, 5, 6 and 8 p.i., in order to evaluate the effect of the two viruses on chicken growth rates.

### Immunosuppressive effects of the GX0101 and GX0101Δ(A+C)

In order to evaluate the immunosuppressive effects of the two viruses, on one-day-old, chickens were inoculated intra-abdominally with 1,000 PFU of GX0101 or GX0101Δ(A+C), while control chickens were inoculated with uninfected CEF cultures. On days 9 p.i., all chickens from each treatment were vaccinated with inactive Newcastle disease virus (NDV), H5 avian influenza viruses (AIV) and H9 AIV. On days 28 p.i., the birds' serums were collected to measure the HI antibody titers to NDV, AIV-H5 and AIV-H9.

## Competing interests

The authors declare that they have no competing interests.

## Authors' contributions

AJS and YPL carried out most of the experiments and wrote the manuscript. JBD and ZZC carried out study design, and revised the manuscript. JYW, SS and HJC helped in vivo experiments, participated data organization and statistical analysis. HFZ helped in revision of the manuscript. All authors read and approved the final manuscript.
